# Brief early antibiotic exposure (≤2 days) is not associated with disruption of gut microbiome in very low birth weight infants

**DOI:** 10.3389/fmicb.2025.1742512

**Published:** 2026-01-14

**Authors:** Yoshinori Aoki, Mayumi Tate, Kayo Ochiai, Kohei Tsuchimochi, Uiko Mizuguchi, Kaoru Okazaki, Hiroshi Moritake

**Affiliations:** 1 Faculty of Medicine, Division of Pediatrics, University of Miyazaki, Miyazaki, Japan; 2 Division of Neonatology, Tokyo Metropolitan Children’s Medical Center, Tokyo, Japan

**Keywords:** antibiotic exposure, antibiotic stewardship, *Bifidobacterium*, gut microbiome, microbial diversity, neonatal intensive care, preterm infants, very low birth weight infants

## Abstract

Early empirical antibiotic therapy is common in preterm and very low birth weight (VLBW) infants but may disrupt the developing gut microbiome. However, the effects of brief antibiotic courses remain unclear, particularly in the most immature infants. In this prospective multicenter cohort study, we examined gut microbiome trajectories in VLBW infants (many of whom were extremely preterm) receiving no antibiotics, a short course (≤2 days), or prolonged exposure (≥3 days). Serial stool samples were analyzed using 16S rRNA gene sequencing. Microbiome composition and diversity in the short-course group were similar to those in unexposed infants at all timepoints, indicating that brief antibiotic exposure did not disrupt microbial development. In contrast, prolonged exposure was associated with transient dysbiosis characterized by reduced *Bifidobacterium* abundance and lower alpha diversity, with partial recovery by discharge. These findings suggest that limiting empirical antibiotic therapy to ≤2 days (48 h) when infection is unconfirmed may not disrupt microbiome development even in highly immature preterm VLBW infants, supporting evidence-based antibiotic stewardship in neonatal intensive care.

## 1 Introduction

Advances in neonatal care have markedly improved the survival of extremely preterm infants (Patel et al., 2015; Pierrat et al., 2017; Cheong et al., 2020). However, infections remain a major cause of morbidity and mortality in this vulnerable population (Weston et al., 2011; Stoll et al., 2015). Among these infections, early-onset sepsis (EOS), which typically presents within the first 72 h of life, is more frequent and has higher fatality rates in preterm infants than in term neonates (Stoll et al., 2011; Shane et al., 2017). Consequently, empirical antibiotic treatment is commonly initiated immediately after birth in preterm infants considered at high risk for EOS, such as those born to mothers with clinical chorioamnionitis, preterm premature rupture of membranes, or other signs of intrauterine infection, even when blood cultures are ultimately sterile (Puopolo et al., 2018).

To ensure clarity and consistency in terminology, we used internationally established definitions: preterm infants as those born at <37 weeks’ gestation (Blencowe et al., 2012), very low birth weight (VLBW) infants as those with a birth weight <1,500 g (World Health Organization, 1992), and extremely preterm infants as those born at <28 weeks’ gestation (Blencowe et al., 2012). Our study population consisted of preterm VLBW infants, the majority of whom were also extremely preterm.

While early empirical antibiotics are often necessary to prevent life-threatening infection, accumulating evidence suggests that they can disrupt the development of the neonatal gut microbiome—a condition known as dysbiosis (Greenwood et al., 2014; Gasparrini et al., 2019). Prolonged antibiotic exposure during this critical developmental period has been associated with increased risks of necrotizing enterocolitis (NEC) and death (Cotten et al., 2009; Alexander et al., 2011). Perturbation of the early microbiome may also impair immune maturation, nutrient absorption, and growth (Gensollen et al., 2016; Uzan-Yulzari et al., 2021) and has been linked to long-term health consequences including gastrointestinal, allergic, metabolic, and neurodevelopmental disorders (Kalliomäki et al., 2008; Vangay et al., 2015; van den Berg et al., 2016; Ihekweazu and Versalovic, 2018).

These concerns have led to increasing emphasis on antibiotic stewardship—defined as the coordinated, evidence-based effort to optimize antibiotic use by ensuring that antibiotics are administered only when necessary, with the appropriate agent, dose, and duration—to minimize unintended harms such as antimicrobial resistance, toxicity, and disruption of the developing microbiome [Centers for Disease Control and Prevention (CDC), 2019].

In recognition of these risks, several international guidelines now recommend discontinuing empirical antibiotics within 36–48 h in infants with suspected EOS if blood cultures remain negative and the clinical course is reassuring [Puopolo et al., 2018; National Institute for Health and Care Excellence (NICE), 2024]. Nevertheless, the degree to which antibiotic exposure alters the neonatal microbiome likely depends on factors such as treatment duration, antibiotic spectrum, and host maturity (Arrieta et al., 2014; Gasparrini et al., 2016; Chang et al., 2021). Broad-spectrum antibiotics, in particular, are thought to have a greater influence on microbial composition (Gibson et al., 2016; Reyman et al., 2022). While longer courses of antibiotics have more profound effects, even short-term antibiotic use may significantly alter gut microbiome (Greenwood et al., 2014; Zwittink et al., 2018). Persistent alterations in microbial communities, including colonization by antibiotic-resistant organisms, have been reported even after discharge (Gasparrini et al., 2019).

A recent study, however, suggested that very short antibiotic exposure—specifically those discontinued within 48 h—had no measurable impact on the gut microbiome of moderately preterm infants with a mean gestational age of about 32 weeks (Kim et al., 2021). However, data remain scarce in extremely preterm and VLBW infants, whose intestinal immaturity, delayed colonization, and limited microbial diversity may render them far more susceptible to antibiotic-induced perturbations.

Therefore, we aimed to determine whether short-course antibiotic exposure—defined as completion by day of life 2—alters gut microbiome development in preterm and VLBW infants. Our cohort included particularly immature infants (median gestational age 27.5 weeks, birth weight 898 g), allowing us to evaluate microbial consequences of antibiotic exposure in one of the most high-risk neonatal populations.

## 2 Materials and methods

### 2.1 Study design and participants

This study was part of a prospective multicenter cohort study initiated in December 2021 and is still ongoing. It is being conducted at two level 3 neonatal intensive care units in Japan: University of Miyazaki Hospital and the Tokyo Metropolitan Children’s Medical Center. The parent study was designed to investigate the development of the neonatal gut microbiome in preterm infants through longitudinal stool sample collection and microbiome analysis.

Eligible participants were preterm and very low birth weight (VLBW) infants, defined as those born at <37 weeks of gestation with a birth weight <1,500 g; those with major congenital anomalies were excluded. In the parent study, stool samples were collected longitudinally starting around the second day of life and continued until discharge.

For the present secondary analysis, we focused on the association between the duration of empirical antibiotic exposure and the composition of the gut microbiome. From the cohort of infants who did not develop gastrointestinal complications, we selected 30 preterm infants, who were subsequently divided into three groups according to the duration of antibiotic administration: None (no antibiotics), Short (≤2 days of age), and Long (≥3 days of age). The cutoff of 2 days for the Short group was chosen to align with clinical guidelines recommending discontinuation within 48 h and to reflect real-world clinical practice (Puopolo et al., 2018). Each group included 10 infants (*n* = 10 per group), comprising 20 infants from the University of Miyazaki Hospital and 10 infants from the Tokyo Metropolitan Children’s Medical Center. A total of 90 stool samples from these 30 infants were analyzed, collected at three predefined time points: week 2 (12–16 days of age), week 4 (26–30 days of age), and at discharge or at 40 weeks’ corrected age. The sample size was determined by the number of eligible infants with complete longitudinal stool sampling available at the time of analysis.

Perinatal and neonatal management decisions were made by the attending physicians. In most cases, prolonged antibiotic administration (Long group) was due to elevated or persistently high infection-related biomarkers such as C-reactive protein, the absence of conclusive culture results, or unstable respiratory conditions that prevented early discontinuation despite negative cultures. Histological chorioamnionitis was diagnosed based on placental pathology (Redline, 2012). For neonates suspected of early-onset sepsis, empirical antibiotic therapy with ampicillin and an aminoglycoside (gentamicin or amikacin) was initiated immediately after birth. Feeding was initiated with maternal breast milk or donor breast milk in most cases. One relatively more mature infant was started on formula feeding.

This study was conducted in accordance with the Declaration of Helsinki, and ethical approval was obtained from the Ethics Committee of the University of Miyazaki Hospital (approval number: O-1053). Written informed consent was obtained from the parents of all participating infants.

### 2.2 Sample collection and DNA extraction

Fecal samples from soiled diapers were collected during hospitalization at week 2 (12–16 days of age), week 4 (26–30 days of age), and at discharge or at 40 weeks’ corrected age. All samples were stored at −80 °C until DNA extraction (Carroll et al., 2012). DNA was extracted using the QIAamp PowerFecal Pro DNA kit (Qiagen, Hilden, Germany) and quantified using a NanoDrop ND-2000 spectrophotometer (Thermo Fisher Scientific, Waltham, MA, USA) according to the manufacturer’s instructions.

### 2.3 16S rRNA gene amplicon sequencing and microbiota analysis

The V3-V4 region of the bacterial 16S rRNA gene was amplified using the following PCR primers: forward primer, 5′-TCGTCGGCAGCGTCAGATGTGTATAAGAGACAGCCTACGGGNGGCWGCAG-3′; reverse primer, 5′-GTCTCGTGGGCTCGGAGATGTGTATAAGAGACAGGACTACHVGGGTATCTAATCC-3′ (Klindworth et al., 2013). Then, the amplicons were sequenced using an Illumina MiSeq platform (Illumina, San Diego, CA, USA).

Sequencing data were processed using QIIME 2 (ver. 2023.9) (Bolyen et al., 2019). Raw sequences were demultiplexed, quality-filtered, and denoised using DADA2 to generate amplicon sequence variants. Taxonomic classification was performed using a classifier pretrained on the SILVA 138 reference database (Quast et al., 2012).

Alpha diversity was assessed using metrics such as the Shannon index and Faith’s phylogenetic diversity. Beta diversity was evaluated based on Bray–Curtis dissimilarity and unweighted UniFrac distances and visualized using principal coordinates analysis (PCoA). To aid interpretation, 95% confidence ellipses representing the distribution of each group were calculated and plotted around the group centroids, using the scikit-bio and matplotlib libraries in Python (ver. 3.11).

### 2.4 Statistical analysis

Continuous variables are expressed as the median and interquartile range (IQR), while categorical variables are expressed as counts with percentages. Alpha diversity indices were compared among the three groups (None, Short, Long) using the Kruskal–Wallis test, followed by Bonferroni-corrected *post hoc* tests when appropriate. At the phylum level, the four most abundant phyla (Actinomycetota, Bacteroidota, Bacillota, and Pseudomonadota), which accounted for nearly all detected sequences, were compared among the three groups using the same statistical approach. At the genus level, *Bifidobacterium*—given its established clinical importance—was analyzed in detail. For visualization and summary reporting, the top 13 genera based on mean relative abundance (including *Bifidobacterium*) were shown in Figure 2A, and their quantitative values and statistical comparisons are provided in Supplementary Table 2. Group differences were evaluated using the same tests. Beta diversity was evaluated based on Bray–Curtis dissimilarity and unweighted UniFrac distances, and statistical comparisons between groups were conducted using PERMANOVA with 999 permutations. For completeness, pairwise PERMANOVA was additionally performed to evaluate differences between individual group pairs. Statistical analyses were performed using SPSS Statistics ver. 28 (IBM Corp., Armonk, NY, USA) and R software ver. 4.2.2 (R Foundation for Statistical Computing, Vienna, Austria). A two-sided *p* value of <0.05 was considered statistically significant.

**Figure 1:**
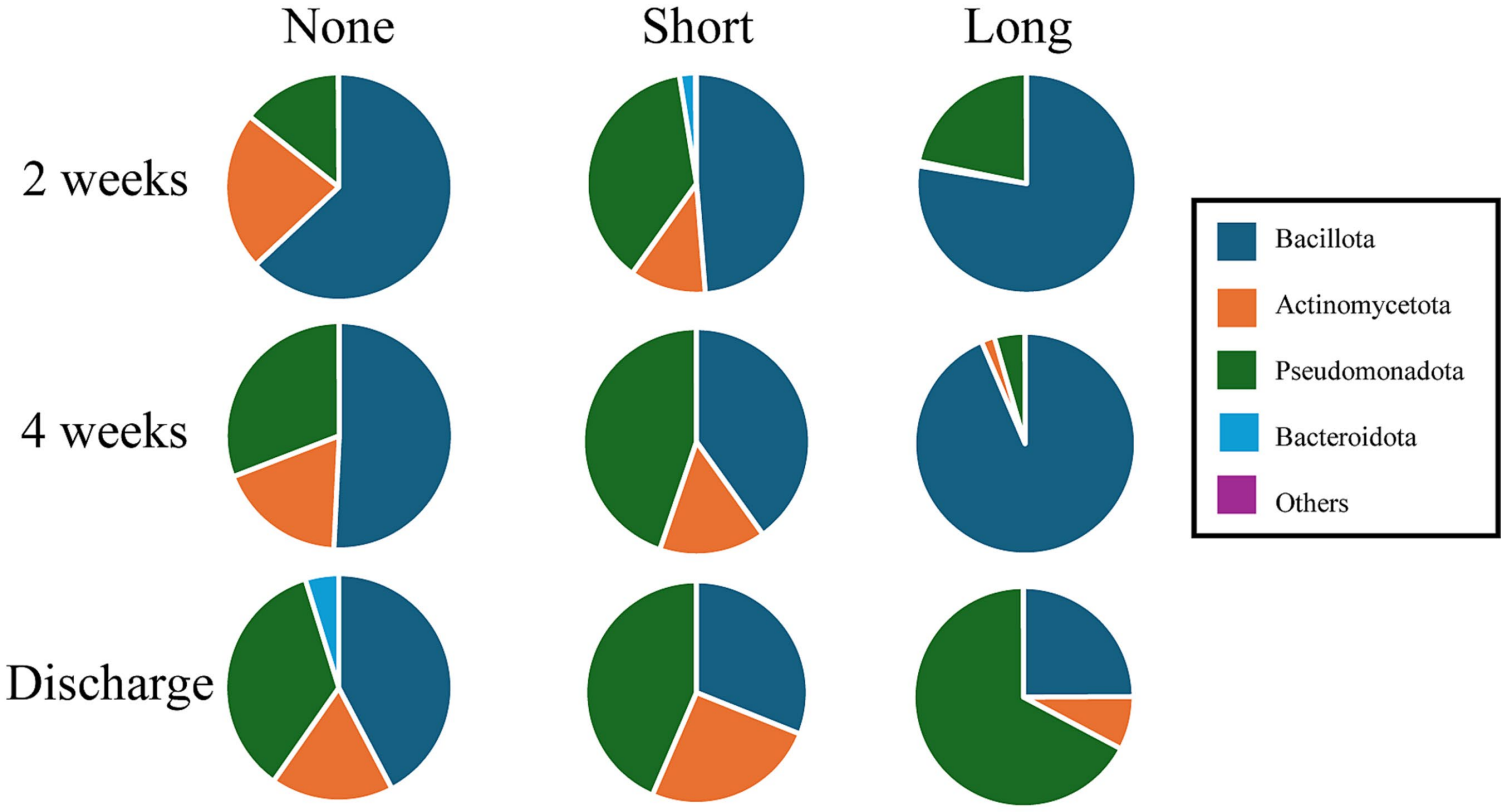
Gut microbiome composition at the phylum level. Relative abundance of gut microbiome at the phylum level at 2 weeks, 4 weeks, and discharge in the three groups (None, Short, and Long). At 2 weeks, Actinomycetota and Bacteroidota differed significantly among groups, while at 4 weeks, significant differences were observed in Bacillota and Pseudomonadota. Longitudinally, the abundance of Actinomycetota and Pseudomonadota increased over time, whereas Bacillota decreased. Statistical comparisons were conducted using the Kruskal–Wallis test with Bonferroni correction; detailed *p*-values are provided in Supplementary Table 1.

## 3 Results

### 3.1 Patient characteristics

The clinical characteristics of the preterm and very low birth weight (VLBW) infants are summarized in Table 1. The participants were categorized into three groups based on the duration of initial antibiotic exposure: None (no antibiotic use), Short (antibiotics ≤2 days), and Long (antibiotics ≥3 days). Overall, the median (IQR) gestational age was 27.5 (25.8, 29.3) weeks, and the median (IQR) birth weight was 898 (680, 1,227) g. Gestational age was significantly lower in the None group [29.5 (28, 33) weeks] compared with the Short group [26 (24.8, 28.3) weeks] and Long group [26 (23.8, 27.3) weeks], with no significant difference between the Short and Long groups. Among the baseline characteristics, gestational age, maternal antibiotic use, and chorioamnionitis showed significant differences across the three groups, whereas none of the other variables differed significantly. Notably, maternal antibiotic use and chorioamnionitis were absent in the None group but were present in both the Short and Long groups, although the difference between the latter two was not significant. Among the infants in the Long group, the main reasons for extended antibiotic use included persistently elevated C-reactive protein or other inflammatory markers, absence of definitive negative culture results, and ongoing respiratory instability.

**Table 1:** Clinical characteristics of the study participants.

Antibiotics	Total (*n* = 30)	None (*n* = 10)	Short (*n* = 10)	Long (*n* = 10)	*p* value
Male sex, *n* (%)	15 (50%)	7 (70%)	2 (20%)	6 (60%)	0.061
Gestational age (weeks), median (IQR)	27.5 (25.8, 29.3)	29.5 (28, 33)	26 (24.8, 28.3)	26 (23.8, 27.3)	0.002
Birth weight (g), median (IQR)	898 (680, 1,227)	1,044 (758, 1,280)	828 (584, 1,228)	886 (641, 1,026)	0.429
Cesarean delivery, *n* (%)	27 (90%)	10 (100%)	8 (80%)	9 (90%)	0.329
Days of initial antibiotics use, median (IQR)	2 (0, 3)	0 (0, 0)	2 (2, 2)	4 (3, 6)	<0.001
Subsequent antibiotics use, *n* (%)	6 (20%)	1 (10%)	3 (10%)	2 (10%)	0.475
Probiotics, *n* (%)	26 (87%)	8 (80%)	9 (90%)	9 (90%)	0.749
Breast milk at 2 weeks, *n* (%)	29 (97%)	10 (100%)	9 (90%)	10 (100%)	0.355
Breast milk at 4 weeks, *n* (%)	25 (83%)	8 (80%)	8 (80%)	9 (90%)	0.787
Breast milk at discharge, *n* (%)	17 (57%)	5 (50%)	4 (40%)	8 (80%)	0.171
Maternal antibiotics, *n* (%)	11 (37%)	0 (0%)	7 (70%)	4 (40%)	0.004
Chorioamnionitis, *n* (%)	12 (40%)	0 (0%)	6 (60%)	6 (60%)	0.007

Comparisons among the three groups were performed using the Kruskal–Wallis test with Bonferroni-corrected post hoc tests. None, no antibiotics; Short, antibiotics for ≤2 days; Long, antibiotics for ≥3 days; IQR, interquartile range.

### 3.2 Gut microbiota composition at the phylum level

The relative abundance of gut microbiota at the phylum level at 2 weeks, 4 weeks, and discharge is shown in Figure 1. The original data and statistical comparisons among the three groups are provided in Supplementary Table 1.

**Figure 2:**
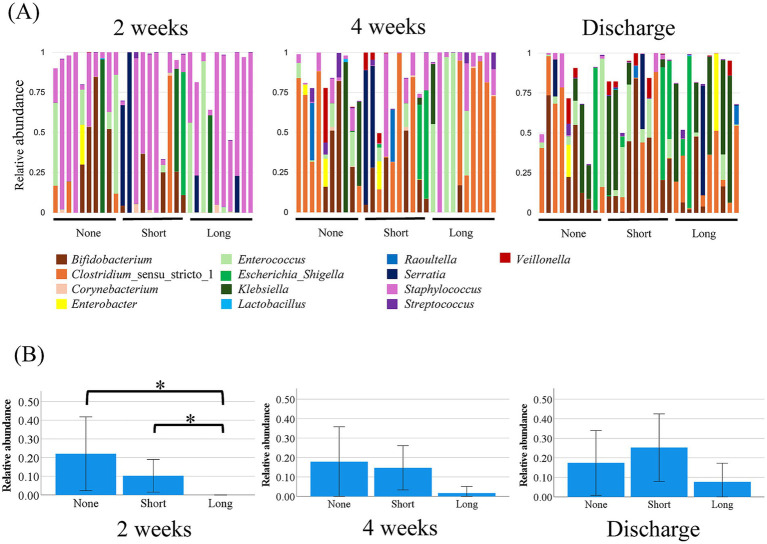
Genus-level microbiome composition and relative abundance of *Bifidobacterium*. **(A)** Genus-level gut microbiome composition at 2 weeks, 4 weeks, and discharge in each group. **(B)** Relative abundance of *Bifidobacterium* at 2 weeks, 4 weeks, and discharge. At 2 weeks, the abundance of *Bifidobacterium* was significantly lower in the Long group compared with the None and Short groups (*p < 0.05; overall *p* = 0.020). At 4 weeks, the Long group continued to show lower values, but the difference was not statistically significant (*p* = 0.118). Differences had diminished by discharge. Statistical comparisons were performed using the Kruskal–Wallis test with Bonferroni correction.

At 2 weeks of age, significant differences were observed in the abundance of Actinomycetota (None 23%, Short 11%, Long 1%; *p* = 0.028) and Bacteroidota (None 0%, Short 2%, Long 0%; *p* = 0.048).

At 4 weeks, Bacillota (None 51%, Short 40%, Long 93%; *p* = 0.006) and Pseudomonadota (None 30%, Short 45%, Long 4%; *p* = 0.024) showed statistically significant differences.

Longitudinally, Actinomycetota and Pseudomonadota increased over time, whereas Bacillota decreased from 2 weeks to discharge.

### 3.3 Genus-level composition and *Bifidobacterium* abundance

Figure 2A shows the genus-level composition of the gut microbiota. Given the well-established importance of *Bifidobacterium* in neonatal gut health (Bäckhed et al., 2015; Underwood et al., 2015; Saturio et al., 2021), we specifically compared its relative abundance among the three groups (Figure 2B).

At 2 weeks of age, the mean relative abundances of *Bifidobacterium* were as follows: None group, 22%; Short group, 10%; and Long group, 0%. There was a significant overall difference among the groups (Kruskal–Wallis test, *p* = 0.020). Pairwise comparisons showed that the abundance of *Bifidobacterium* was significantly lower in the Long group compared with the None group (*p* = 0.042) and the Short group (*p* = 0.048).

At 4 weeks of age, the mean relative abundances were as follows: None group, 17%; Short group, 15%; and Long group, 2%. The Short group did not differ from the None group, while the abundance in the Long group was lower but this difference did not reach statistical significance (*p* = 0.118).

In addition to *Bifidobacterium*, we explored the dynamics of other early-colonizing genera with relatively high abundance. The full genus-level quantitative results and statistical comparisons are provided in Supplementary Table 2. At discharge, the relative abundance of *Staphylococcus* differed among the three groups (None 3.3%, Short 1.6%, Long 0.2%; *p* = 0.044), although subsequent pairwise comparisons did not reveal significant differences. No other genera exhibited consistent or notable group-related differences.

By the time of discharge, the differences among the groups had further diminished, suggesting partial recovery of *Bifidobacterium* colonization over time.

### 3.4 Beta diversity analysis

**Figure 3:**
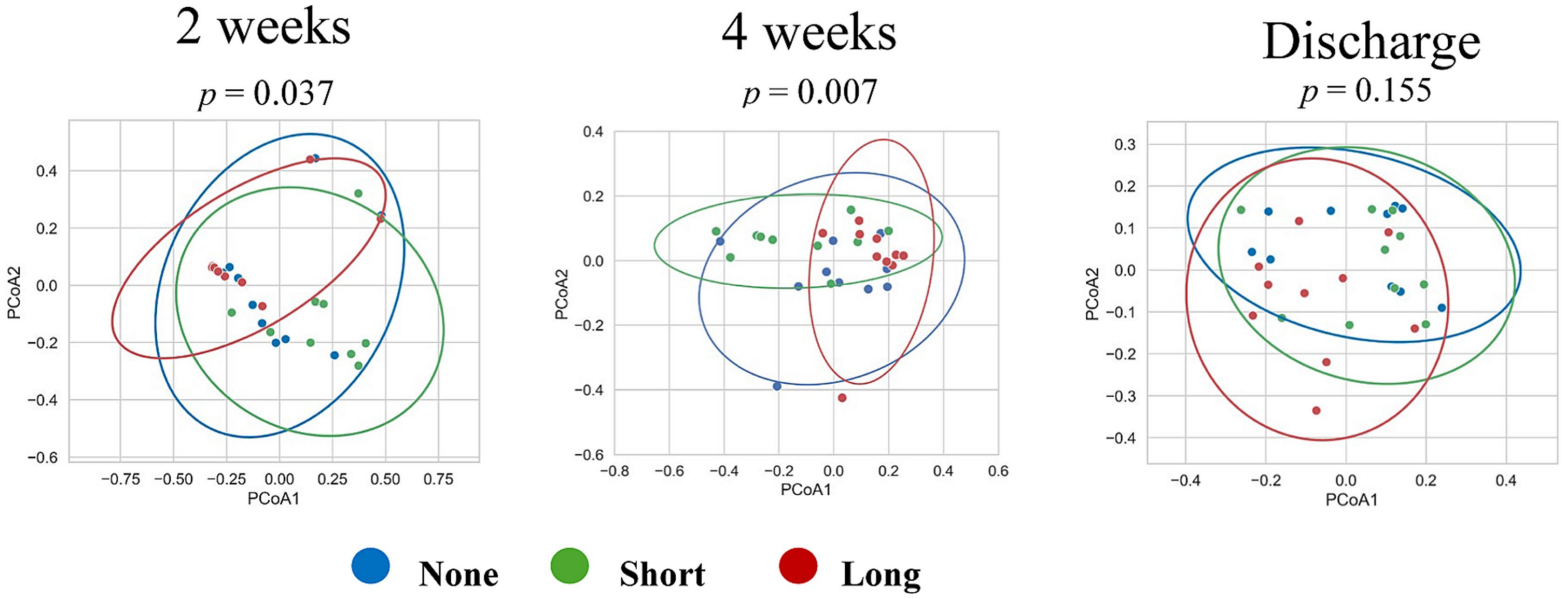
Beta diversity of gut microbiome. Beta diversity was evaluated using Bray–Curtis dissimilarity and unweighted UniFrac distances, and visualized by principal coordinates analysis (PCoA) at 2 weeks, 4 weeks, and discharge. Colored ellipses represent 95% confidence intervals for each group, calculated from the PCoA coordinates using Python. At 2 weeks, overall group differences were significant (*p* = 0.037), with a significant difference between the Short and Long groups (*p* = 0.018). At 4 weeks, differences became more pronounced (overall *p* = 0.007), with pairwise differences between the None and Long groups (*p* = 0.037) and between the Short and Long groups (*p* = 0.002). No significant difference was found between the None and Short groups. By discharge, group differences were no longer observed. Statistical comparisons were performed using PERMANOVA.

Beta diversity was evaluated using Bray–Curtis dissimilarity and unweighted UniFrac distances, and visualized PCoA (Figure 3). For visualization, 95% confidence ellipses were overlaid for each group to illustrate the distribution and separation of microbial communities. The original data, showing PCoA1 and PCoA2 values for all 30 samples, are provided in Supplementary Table 3.

At 2 weeks of age, a significant difference in overall community structure was observed among the three groups (*p* = 0.037), indicating that they formed distinct microbial communities. Pairwise comparison showed that the Short and Long groups differed significantly (*p* = 0.018), while no significant difference was observed between the other pairs.

At 4 weeks of age, the separation between the Long group and the other groups became more pronounced. Overall group differences were significant (*p* = 0.007), and pairwise comparisons revealed significant differences between the None and Long groups (*p* = 0.037), and between the Short and Long groups (*p* = 0.002). There was no significant difference between the None and Short groups.

By the time of discharge, these differences were no longer observed.

### 3.5 Alpha diversity

**Figure 4:**
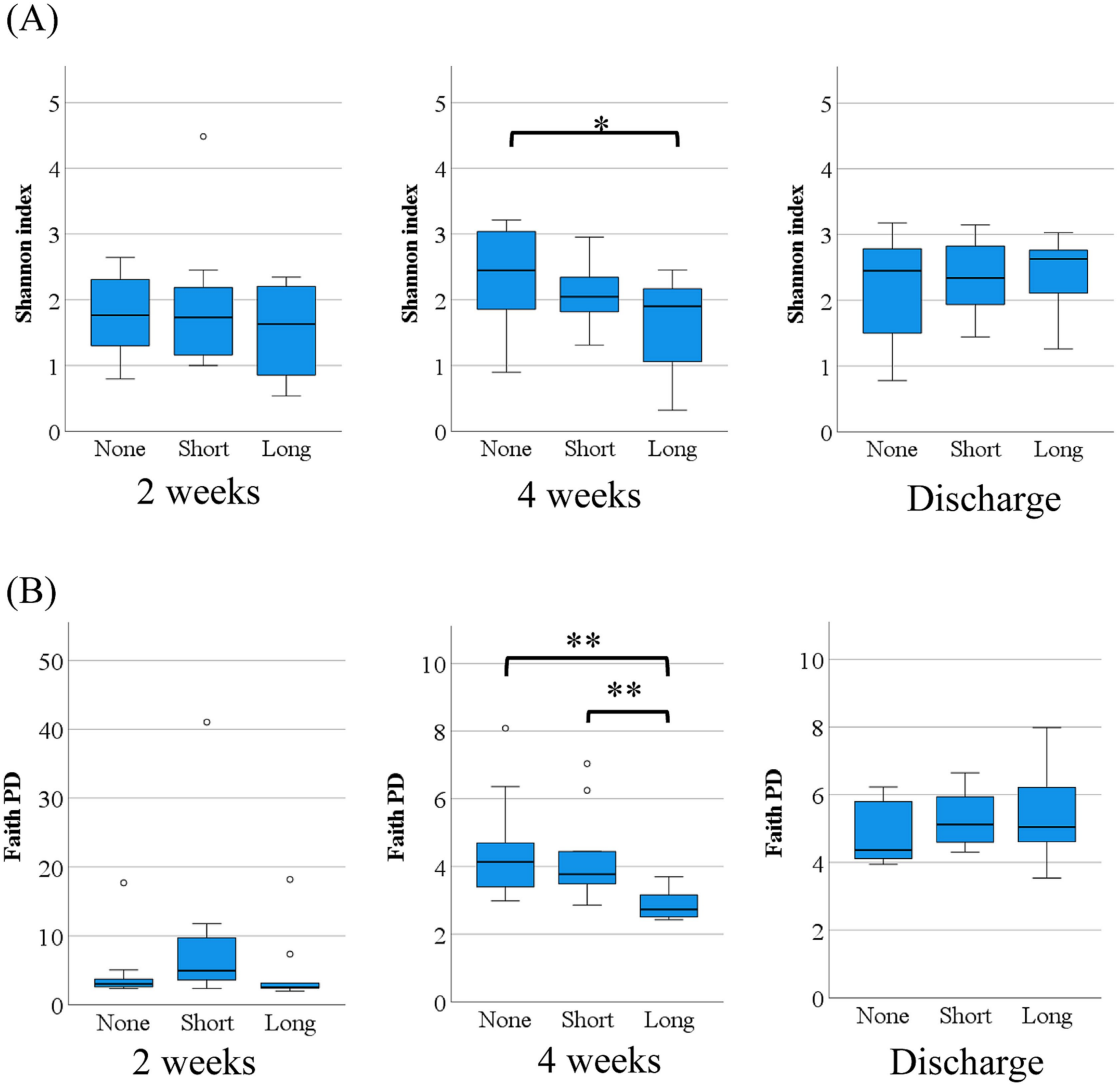
Alpha diversity of gut microbiome. **(A)** Shannon diversity index and **(B)** Faith’s phylogenetic diversity at 2 weeks, 4 weeks, and discharge. At 4 weeks, both indices showed significantly lower alpha diversity in the Long group compared with the None and Short groups (*p < 0.05, **p < 0.01). At discharge, no significant differences in alpha diversity were observed among the groups for either index, suggesting partial recovery of diversity over time. Statistical comparisons were performed using the Kruskal–Wallis test with Bonferroni correction.

Figure 4 shows the alpha diversity evaluated according to the Shannon diversity index (Figure 4A) and Faith’s phylogenetic diversity (Figure 4B).

At 4 weeks, the Long group exhibited significantly lower Shannon diversity compared with the None group (mean 1.6 vs. 2.3, *p* = 0.041), while the Short group (mean 2.1) showed no significant difference compared with the None group. Similarly, Faith’s phylogenetic diversity was significantly lower in the Long group compared with both the None group (mean 2.9 vs. 4.5) and the Short group (mean 2.9 vs. 4.2, *p* < 0.01 for both), indicating a marked reduction in phylogenetic richness associated with prolonged antibiotic exposure.

At the time of discharge, no significant differences in alpha diversity were observed among the three groups for either metric, suggesting a partial recovery or convergence of microbial diversity over time.

## 4 Discussion

In this study, we investigated the impact of early antibiotic exposure on the gut microbiome development in preterm and very low birth weight (VLBW) infants, most of whom were extremely preterm (median gestational age 27.5 weeks, birth weight 898 g). We found that brief antibiotic exposure (≤2 days) did not alter the composition or diversity of the gut microbiome at 2 and 4 weeks of age compared with infants who did not receive antibiotics. In contrast, prolonged exposure (≥3 days, median 4 days) was associated with reduced *Bifidobacterium* abundance and lower microbial diversity at the same time points. These differences had diminished by discharge, suggesting a partial recovery of the gut microbiome over time. Collectively, our findings indicate that antibiotic duration determines whether measurable disruption occurs, with brief exposure showing no detectable alteration of gut microbiome development. Furthermore, our study provides insights into the effects of early antibiotic exposure, specifically in extremely preterm infants, a population that has been less well characterized in previous research.

Our findings align in part with previous studies that demonstrated significant disruptions of the neonatal gut microbiota following antibiotic administration. Prolonged exposure in preterm infants has been associated with reduced microbial diversity, delayed colonization by commensal bacteria such as *Bifidobacterium*, and increased risks of NEC and late-onset sepsis (Cotten et al., 2009; Greenwood et al., 2014; Pammi et al., 2017; Zwittink et al., 2018; Chang et al., 2021). These alterations can persist for weeks or even months after treatment (Tanaka et al., 2009; Yassour et al., 2016; Gasparrini et al., 2019).

Evidence on the impact of short antibiotic courses is limited. The only study to specifically address this issue, to our knowledge, reported that antibiotic exposure limited to ≤48 h had no measurable impact on microbial composition compared with unexposed infants (Kim et al., 2021). Importantly, that study population (mean gestational age, about 32 weeks) was more mature than ours, raising uncertainty about whether the same applies to extremely preterm infants. Notably, our cohort was considerably less mature and included many extremely preterm infants (<28 weeks), a population with delayed intestinal maturation, limited microbial resilience, and heightened vulnerability to external perturbations. The fact that we similarly observed no disruption at ≤2 days of exposure strengthens the conclusion that brief administration of empirical antibiotics, even in extremely preterm infants, does not cause lasting microbial disturbance.

In contrast, our findings in the Long group (≥3 days, median 4 days) consistent with prior studies demonstrating measurable dysbiosis after extended antibiotic exposure (Greenwood et al., 2014; Zwittink et al., 2018; Chang et al., 2021). However, unlike some reports describing persistent disturbances weeks or months after exposure (Tanaka et al., 2009; Yassour et al., 2016; Gasparrini et al., 2019), we found that microbial composition had converged by discharge. The relatively short duration of treatment in our cohort—typically 4 days, shorter than in many earlier studies where antibiotic courses often exceeded 7–10 days—may partly explain this recovery. These results support a duration-dependent relationship between antibiotic exposure and gut microbiome disruption: while exposure ≤48 h appears to have minimal effects, extending treatment beyond this threshold can induce measurable, though transient, alterations.

Mechanistically, antibiotics likely disrupt gut microbial development through depletion of key pioneer taxa such as *Bifidobacterium*, which play critical roles in shaping early intestinal ecology and promoting mucosal immune maturation (Saturio et al., 2021; Lin et al., 2022;). Their depletion may lead to reduced microbial diversity and delayed establishment of a stable community (Saturio et al., 2021; Turroni et al., 2022). Conversely, the limited effect of brief exposure (≤2 days) may reflect the rapid restoration of this pioneer genus once antibiotic pressure is removed. These findings suggest that the neonatal gut ecosystem has a degree of resilience to short perturbations, whereas prolonged antibiotic pressure may exceed this compensatory capacity, resulting in transient dysbiosis.

Our results have important clinical implications for neonatal care. Empirical antibiotics remain indispensable in preterm infants at high risk for EOS, where the consequences of delayed or missed treatment can be fatal. However, unnecessary prolongation of antibiotics can disrupt the fragile process of gut microbial colonization, with potential downstream effects on immunity, metabolism, and neurodevelopment. Our findings suggest that discontinuing antibiotics within 36–48 h, as recommended by current guidelines when cultures are negative and the infant remains clinically stable [Puopolo et al., 2018; National Institute for Health and Care Excellence (NICE), 2024], is unlikely to cause measurable disruption of the gut microbiome, even in extremely preterm infants. In contrast, extending treatment beyond this period (≥3 days) was associated with detectable, though reversible, alterations. These results reinforce the importance of limiting antibiotic duration to the minimum necessary for safety, thereby balancing the benefits of infection control with the preservation of early microbial development.

Ultimately, decisions about antibiotic use in preterm infants must balance the clear benefits of infection control against the potential risks of dysbiosis. The present study provides microbiome-based evidence supporting the safety of brief empirical antibiotic use and highlights that timely discontinuation of empirical antibiotics preserves gut microbiome development without compromising safety.

### 4.1 Strengths and limitations

The strengths of our study include its focus on extremely preterm infants, who are underrepresented in prior research, and the longitudinal assessment of gut microbiome at multiple time points until discharge. This design allowed us to capture both early disruptions and subsequent recovery patterns.

Several limitations should also be acknowledged. First, the sample size was modest, limiting statistical power and the ability to adjust for all confounders. However, although some perinatal factors such as gestational age, maternal antibiotic use, and chorioamnionitis differed among the three groups, these variables did not differ significantly between the Short and Long groups. Moreover, despite the less favorable perinatal conditions in the Short group compared with the None group—conditions generally associated with negative microbiome effects—their microbial profiles were largely comparable. These findings suggest that the observed differences were primarily related to antibiotic duration rather than baseline disparities.

Second, analyses were based on 16S rRNA gene sequencing rather than shotgun metagenomics, restricting taxonomic resolution and functional insights.

Third, because of the small number of infants, we were unable to evaluate whether differences in antibiotic exposure influenced the occurrence of NEC, sepsis, or other morbidities.

In addition, antibiotic duration was determined by the attending physicians based on clinical and laboratory findings rather than by protocol, indication bias cannot be entirely excluded.

Finally, our analysis was limited to the hospitalization period; therefore, the longer-term consequences of early antibiotic exposure could not be assessed.

## 5 Conclusion

Brief empirical antibiotic exposure (≤2 days) did not alter gut microbiome development in preterm and very low birth weight (VLBW) infants, whereas prolonged exposure (≥3 days, median 4 days) was associated with transient dysbiosis, characterized by reduced *Bifidobacterium* abundance and lower microbial diversity at 2–4 weeks of age. These findings highlight the importance of minimizing unnecessary antibiotic use and support guideline-recommended discontinuation within 48 h when sepsis is not confirmed. Future studies with larger cohorts, extended longitudinal follow-up, and metagenomic approaches are needed to clarify the functional and long-term consequences of early-life dysbiosis and identify strategies that promote optimal microbiome development in this vulnerable population.

## Data Availability

The data presented in this study are publicly available. The data can be found here: https://www.ncbi.nlm.nih.gov, accession PRJNA1346831.
